# Molecular characterization of a new R1925X point mutation mouse model for dysferlinopathy

**DOI:** 10.1016/j.gendis.2025.101885

**Published:** 2025-10-20

**Authors:** Camille Bouchard, Louis Despax, Joël Rousseau, Jacques P. Tremblay

**Affiliations:** a Université Laval, Département de Médecine moléculaire, Québec G1V 4G2, Canada; b Centre de recherche du Centre Hospitalier Universitaire de Québec, Québec G1V 4G2, Canada; c Université de Montpellier, Faculté de Pharmacie, Montpellier 14491 34093, France

Dysferlinopathy affects between one person in 14 000 to one in two million.^1^ It is caused by mutations in the DYSF gene, a 55-exon gene on chromosome 2p13.^2^ Dysferlin is a 237 kDa membrane protein, which plays a role in stabilizing Ca^2+^ signalization and repairing the sarcolemma in skeletal muscles.^3^ Mutations in the DYSF gene cause a progressive muscle-wasting disease called dysferlinopathy. This disease has no approved treatment yet and induces progressive skeletal muscle atrophy. The age of onset is 20 ± 5 years, and patients notice weakness and atrophy in the calf or thigh, which evolves to include the upper limbs.^4^ The typical signs and symptoms are muscle wasting (27%), pain, stiffness, or cramps (13%), or pseudohypertrophy (6%), and elevated blood creatine kinase (CK) levels.^4^ The patients, therefore, need to walk with a cane in their thirties and become wheelchair bound in their fourth decade of life. Among the affected patients, several have point mutations (nonsense or missense). Therefore, having a mouse model with a point mutation is pertinent for developing a therapy to correct mutations (see Fig. 1).

**Figure 1:**
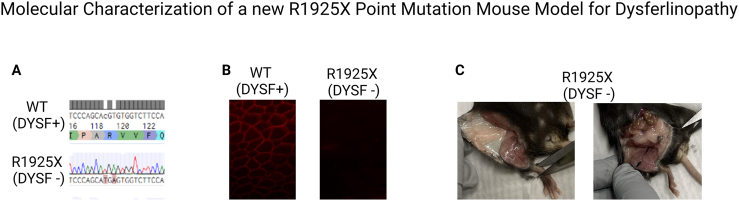
Molecular characterization of a new R1925X point mutation mouse model for dysferlinopathy. **(A)** The CGT(R) to TGA (stop) mutation is present in all R1925X mice. **(B)** C57BL6 mice were used for the wild-type control and R1925X mice. Immunohistochemistry using the Ab Cam JAI-1-49-3 (Romeo) antibody coupled with Alexa Fluor 546 goat anti-rabbit IgG H + L antibody was performed on 4 wild-type tibialis anterior (TA) muscles and 12 R1925X TA muscles. All wild-type muscles showed red staining around the fibres, and all R1925X muscles showed no significant staining. **(C)** A lipid accumulation was noticed in the lower abdomen and upper leg regions of all 3 DYSF-R1925X female mice during dissection.

Many dysferlin knockout mouse models are available to study the impacts of molecular treatments on dysferlinopathy. However, a mouse model with a mutation similar to a mutation present in human patients with dysferlinopathy is still needed to study the impact of gene therapy, especially gene editing therapy, on the disease. A new mouse model with a nonsense point mutation in position R1925X of the DYSF gene, corresponding to the human R1905X mutation, is now available from the Jain Foundation and could be used to evaluate the impact of gene therapy on the phenotype. This specific mutation was chosen because it is a patient mutation and allows for testing of gene editing, such as Prime editing. We verified that the DYSF-R1925X mouse model has the desired mutation. We did so by amplifying a 322 bp region around the mutation and using Sanger sequencing. The amplified section was indeed of the desired size, and the sequencing showed the presence of the CGT to TGA mutation in all mice. We also confirmed the absence of dysferlin expression in muscles by immunohistochemistry and Western blotting. During the mouse dissection, we also noticed a fat accumulation in the lower abdomen and upper leg region in female R1925X mice.

The mutation DYSF-R1925X has been inserted by Prime editing *in utero* by the Jain Foundation/Jackson Laboratory (USA). We verified that this mutation was correctly inserted by amplifying a 322 bp region around the mutation (Fig. S1), followed by Sanger sequencing. The amplified section was indeed of approximately 300 bp (Fig. S2), and the sequencing showed the presence of the CGT to TGA mutation in all 6 mice (Fig. S3). We also confirmed the absence of dysferlin expression in muscles by immunohistochemistry (Fig. S4) and Western blotting (Fig. S5). The wild-type control presented a 250 kDa band, which is the complete dysferlin protein. It also contained a 150 kDa and a smaller fragment that correspond to the cleaved dysferlin (Fig. S6). In fact, we used the Romeo antibody, which targets the N-terminal region of the dysferlin protein. The 150 kDa band is complementary to the 72 kDa band detected in the C-terminal by the Hamlet antibody.^5^ During the mouse dissection, we also noticed a fat accumulation in the lower abdomen and upper leg region in female DYSF-R1925X mice (Fig. S7). The National Center for Biotechnology Information by the National Library of Medicine shows that dysferlin is highly expressed in the genital fat pad and subcutaneous fat pad in adult mice (RPKM of 9.2 and 7.4) (https://www.ncbi.nlm.nih.gov/gene/26903). It is also comparably expressed in human fat (RPKM of 5.5 ± 3.4) (https://www.ncbi.nlm.nih.gov/gene/8291).

The DYSF-R1925X mouse is a newly designed mouse model for dysferlinopathy inspired by the human R1905X mutation in the DYSF gene. It was not characterized in literature yet, and we confirm the presence of the R1925X mutation in the mouse DYSF gene as well as the absence of dysferlin expression in the tibialis anterior muscles. We also noticed fat accumulation in the female lower abdomen and thighs. These mice are therefore suitable to study the impact of gene therapy to correct point mutations in the DYSF gene. The next step will be to characterize the phenotype, including muscle strength and composition, as well as the mice's general behavior.

## CRediT authorship contribution statement

**Camille Bouchard:** Writing – review & editing, Writing – original draft, Methodology, Funding acquisition, Formal analysis, Data curation, Conceptualization. **Louis Despax:** Writing – original draft, Formal analysis, Data curation. **Joël Rousseau:** Conceptualization. **Jacques P. Tremblay:** Supervision, Project administration, Funding acquisition, Conceptualization.

## Ethics declaration

All experiments involving animals were approved by the animal care committee of the Hospital Center of Laval University (CHU de Québec-Université Laval; Québec, Canada).

## Funding

This research received funding from the Jain Foundation (Seattle, Washington, USA) (No. TREM-01).

## Conflict of interests

The authors declared no conflict of interests.
