# Supplementary material for: Molecular characterization of a new R1925X point mutation mouse model for dysferlinopathy
Source: Genes Dis. 2025 Oct 20;13(3):101885. doi: 10.1016/j.gendis.2025.101885 (PMC12824904; doi:10.1016/j.gendis.2025.101885)
Supplement: Multimedia component 1 [file mmc1.docx]

**Material and Methods**

Mouse housing

The DYSF-R1925X mice were reproduced in the Jackson Laboratory facility. The mice were housed 2–4 per standard ventilated cage under a 12-h light/dark cycle with access to food and water ad libitum. Neslet and Aspen shaving are given for nidification. All experiments involving animals were approved by the animal care committee of the Hospital Center of Laval University (CHU de Québec-Université Laval; Québec, Canada). The wildtype mice are C57BL6 mice reproduced in our animal facility and housed under the same conditions.

DNA extraction

DNA from cells was extracted by resuspending the cells in 100 µL DirectPCR Lysis Reagent (Tail): (Viagen, Los Angeles, USA) with 0.02 ng Proteinase K (Sigma, Burlignton, USA) and incubating for 2h hours at 56°C, then inactivating the Proteinase K for 45 minutes at 85°C.

Primer design

The PCR primers were designed to amplify a 322 bp fragment around the R1925X mutation in the DYSF gene. A forward primer was also designed for Sanger sequencing of the previously amplified fragment.

PCR primers : F : GGCTGGATGGTGGGATTC R : TGCTCAAAAAGGGACACAAACC

Sanger sequencing Forward internal primer : GCAGAAGACAGATGTGCACTACC

PCR

A PCR was performed using 1 µL of the previoulsy extracted DNA along with 10 µL G5X buffer, 1 µL dNTPs 10 µM, 1 µL Forward primer (10 µM), 1 µL Reverse primer (10 µM), 0.25 µL Phusion enzyme and completing to 50 µL per tube with DNAse-free water. The PCR was performed in the thermocycler with the following settings:


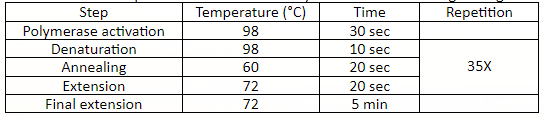


Sanger Sequencing

The Sanger sequencing is done by the Plateforme de Séquençage du Centre de Recherche du Centre Hospitalier Universitaire de Québec – Université Laval SANGER Sequencing – Research Center (ulaval.ca).

Euthanasia

The mice were sacrificed with CO2 under isoflurane anesthesia at around 26 weeks. The Tibialis anterior muscle was removed and frozen in OCT with liquid nitrogen before being stored at −80◦C until analysis.

Immunohistochemistry

After sacrifice, the Tibialis anterior muscles from DYSF-R1925X mice and 2 control mice were harvested. Muscles were embedded in Optimal Cutting Temperature Embedding Medium (OCT compound) (Fisher Healthcare, Houston, USA) to make 32 μm thick slides with a cryostat. Slides were washed with phosphate-buffered saline (PBS) before reaction. Slides were saturated with a mixture of goat serum and fetal bovine serum for 30 min. Slides were then incubated with a 500 fold dilution of rabbit anti-dysferlin antibody (Ab Cam JAI-1-49-3 Antibody) in the presence of fetal bovine serum for 1.5 h. Slides were washed 3 times with PBS before incubation for 1.5 h with the fluorophore-coupled Alexa Fluor 546 goat anti-rabbit IgG H+L antibody (invitrogen, Eugene, USA). Slides were washed 3 times with PBS, then mounted with 50% Glycerol, 50% PBS for observation under a red fluorescence light microscope (x40).

Western blot

Total proteins were extracted from mouse Tibialis anterior muscles. Muscles included in the OCT were washed in PBS before being crushed in an aqueous extraction solution: 2% Tris/HCl(1 M; pH7.5); 0.1% Dithiothreitol (100 mM); 1% Phenylmethylsulfonyl fluoride; 5% Sodium dodecyl sulfate. Then 600 μL of methanol, 200 μL of chloroform and 500 μL of H2O were added to 200 μL of supernatant from the crush. The mixture was vortexed and centrifuged for 5min at maximum speed (13 000 rpm). The white interface formed was recovered and washed with 300 μL of methanol. The interface was then dried and 100 μL of a solution of Laemmli blue and Mercaptoethanol was added. The solution is boiled for 5 min at 95°C. Total proteins were then quantified using the “Qubit™ Protein Assay” protocol. 20 μg of protein were deposited in each well of an SDS-Page gel (8% polyacrylamide; Tris/HCl (pH8.8/1.5 M)). The molecular weight marker used was “Precision Plus Protein™” from BioRad (Hercules, USA). Electrophoresis was conducted with a voltage of 100 V for a duration of 1.75 h. The gel was then transferred to a polyvinylidene fluoride (PVDF) membrane. The membrane was then saturated with milk proteins and incubated with 5 μL of anti-dysferlin antibody (Ab Cam JAI-1-49-3 antibody) overnight. The membrane was washed 3 times for 10 minutes with a washing solution (PBS; 5%Polysorbate 20). The membrane was then incubated with 2 μL of anti-rabbit horseradish peroxidase (HRP) in the presence of milk protein for 2 h. The membrane was washed 3 times with the washing solution. 1 mL of a solution of hydrogen peroxide (50%) and luminol (50%) is deposited on the membrane to reveal chemiluminescence.
