# Supplementary material for: Molecular characterization of a new R1925X point mutation mouse model for dysferlinopathy
Source: Genes Dis. 2025 Oct 20;13(3):101885. doi: 10.1016/j.gendis.2025.101885 (PMC12824904; doi:10.1016/j.gendis.2025.101885)
Supplement: Multimedia component 2 [file mmc2.docx]

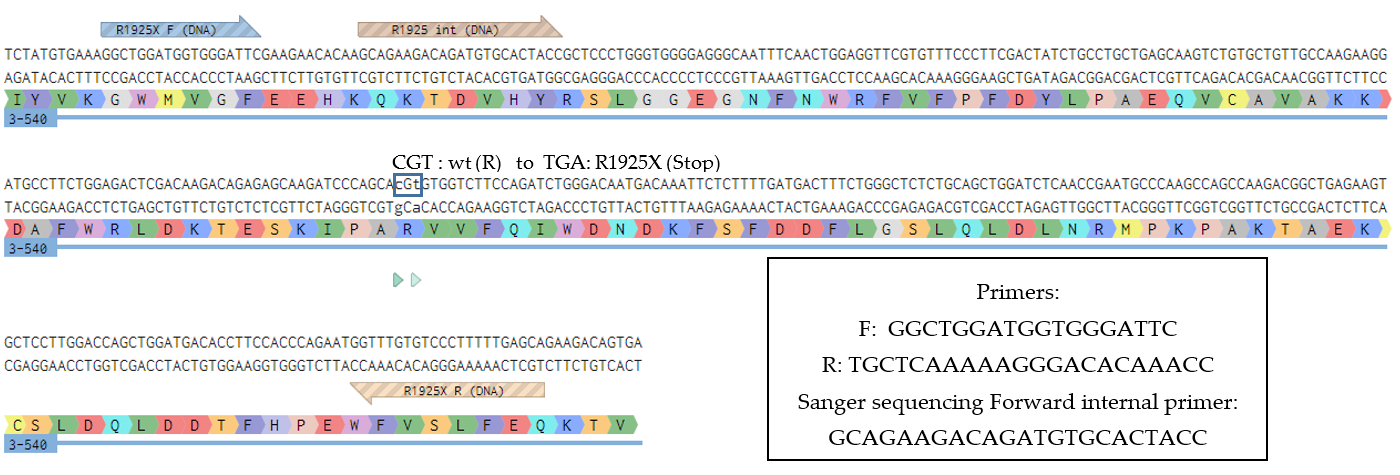


**Supplementary Figure 1: Map of the mouse mutation with PCR and sequencing primers**. The wildtype arginin (R) in position 1925 is encoded by CGT nucleotides in mice. The mutation of two nucleotides in mice is TGA, causing a premature stop codon equivalent to the human R1905X mutation. Primers were designed to amplify a 322 bp region around the mutation by PCR and a forward primer for Sanger sequencing was designed inside the the amplified region.

PCR gene amplification (322 bp)

1 2 3 4 5 6 7


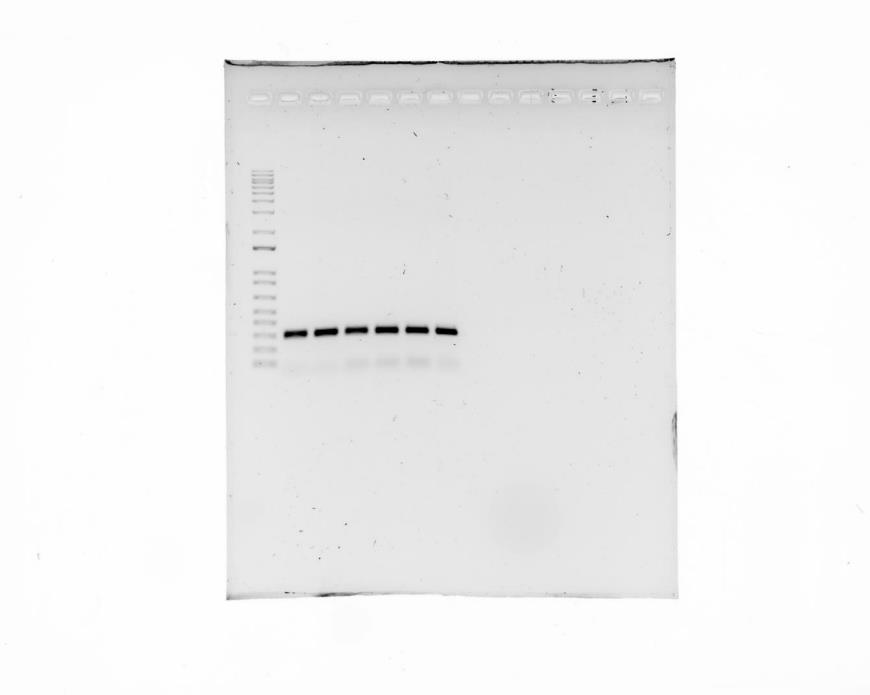


300 bp

**Supplementary Figure 2: PCR gene amplification (322 bp).** The 322 bp region was amplified by PCR and used for sequencing. Well 1 : 1kb DNA ladder. Wells 2 to 7, R1925X mice 1 to 6.


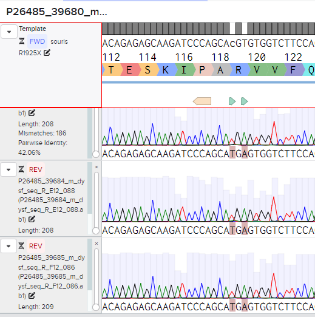


**Supplementary Figure 3:** **Sanger sequencing of the R1925X mutation area.** The Sanger sequencing results alignement on Benchling shows that the TGA (stop) mutation is present in all R1925X mice. The wildtype mice have a CGT (arginine) genotype.


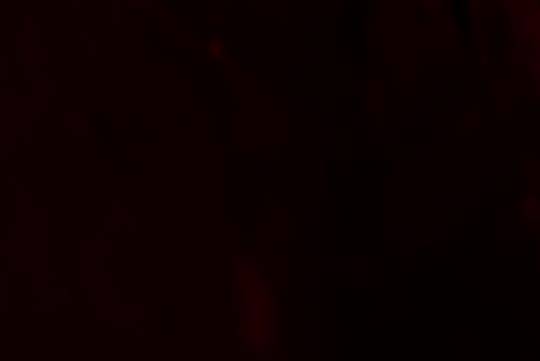

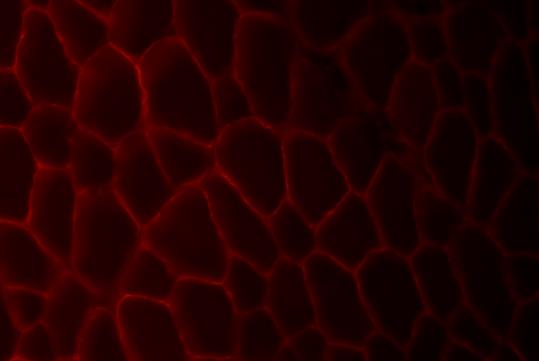
 WT (DYSF +) R1925X (DYSF -)

**Supplementary Figure 4: Immunohistochemistry (IHC) : Ab Cam JAI-1-49-3 Antibody (Romeo).** C57BL6 mice were used for the wildtype control and R1925X mice. Immunohistochemistry using the Ab Cam JAI-1-49-3 (Romeo) antibody coupled with Alexa Fluor 546 goat anti-rabbit IgG H+L antibody was performed on 4 wild type *Tibialiss anterior* (TA) muscles and 12 R1925X TA muscles. All WT muscles showed red staining around the fibres and all R1925X muscles showed no significant staining.


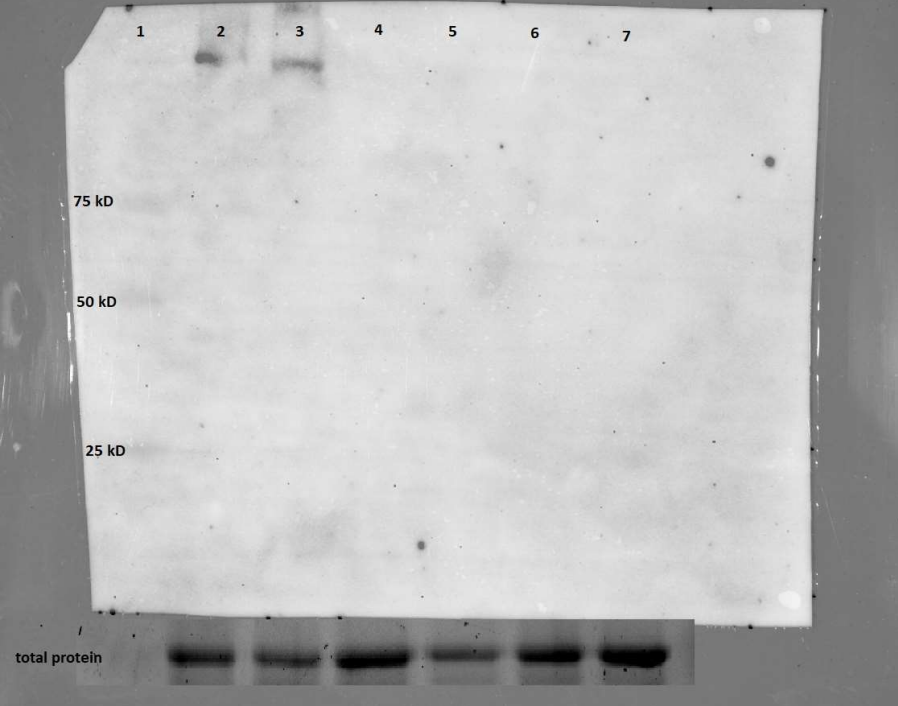


**Supplementary Figure 5: Western blot: Ab Cam JAI-1-49-3 Antibody (Romeo).** C57BL6 mice were used for the wild type control and R1925X mice. Western blot using the Ab Cam JAI-1-49-3 (Romeo) antibody coupled with anti-rabbit horseradish peroxidase (HRP) was performed on 2 wild type *Tibialis anterior* (TA) muscles and 5 R1925X TA muscles. The wild type muscles showed a 250 kDa band and all R1925X muscles showed no significant band.


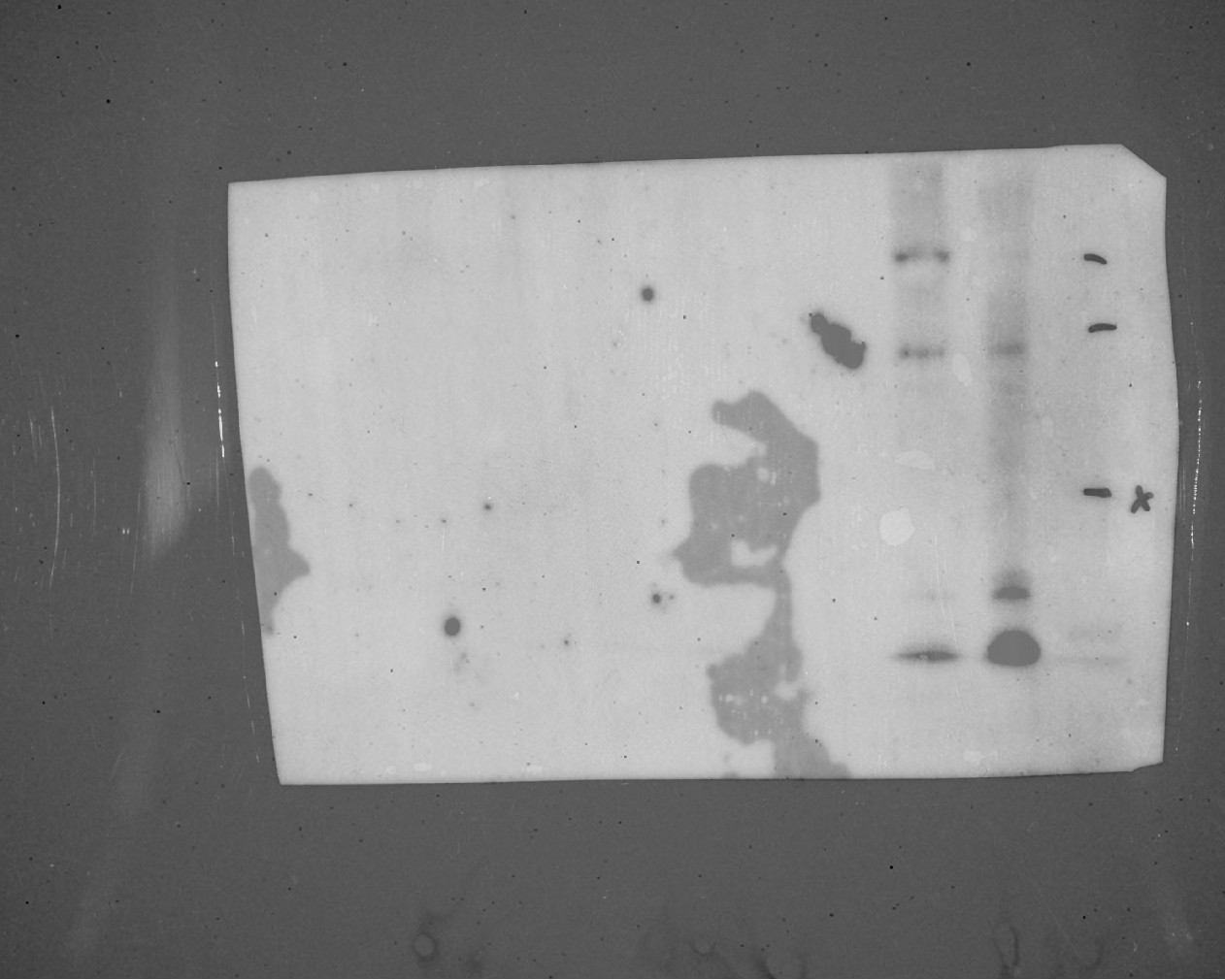


R1925X R1925X R1925X R1925X R1925X R1925X WT


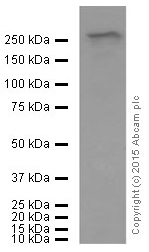

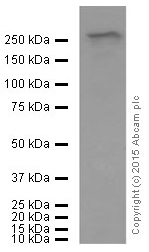

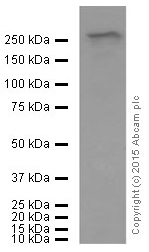


**Suplementary Figure 6: Western blot: Ab Cam JAI-1-49-3 Antibody (Romeo).** C57BL6 mice were used for the wild type control and R1925X mice. Western blot using the Ab Cam JAI-1-49-3 (Romeo) antibody coupled with anti-rabbit horseradish peroxidase (HRP) was performed on 1 wild type *Tibialis anterior* (TA) muscles and 6 R1925X TA muscles. The wild type muscle showed a 250 kDa band and all R1925X muscles showed no significant band.


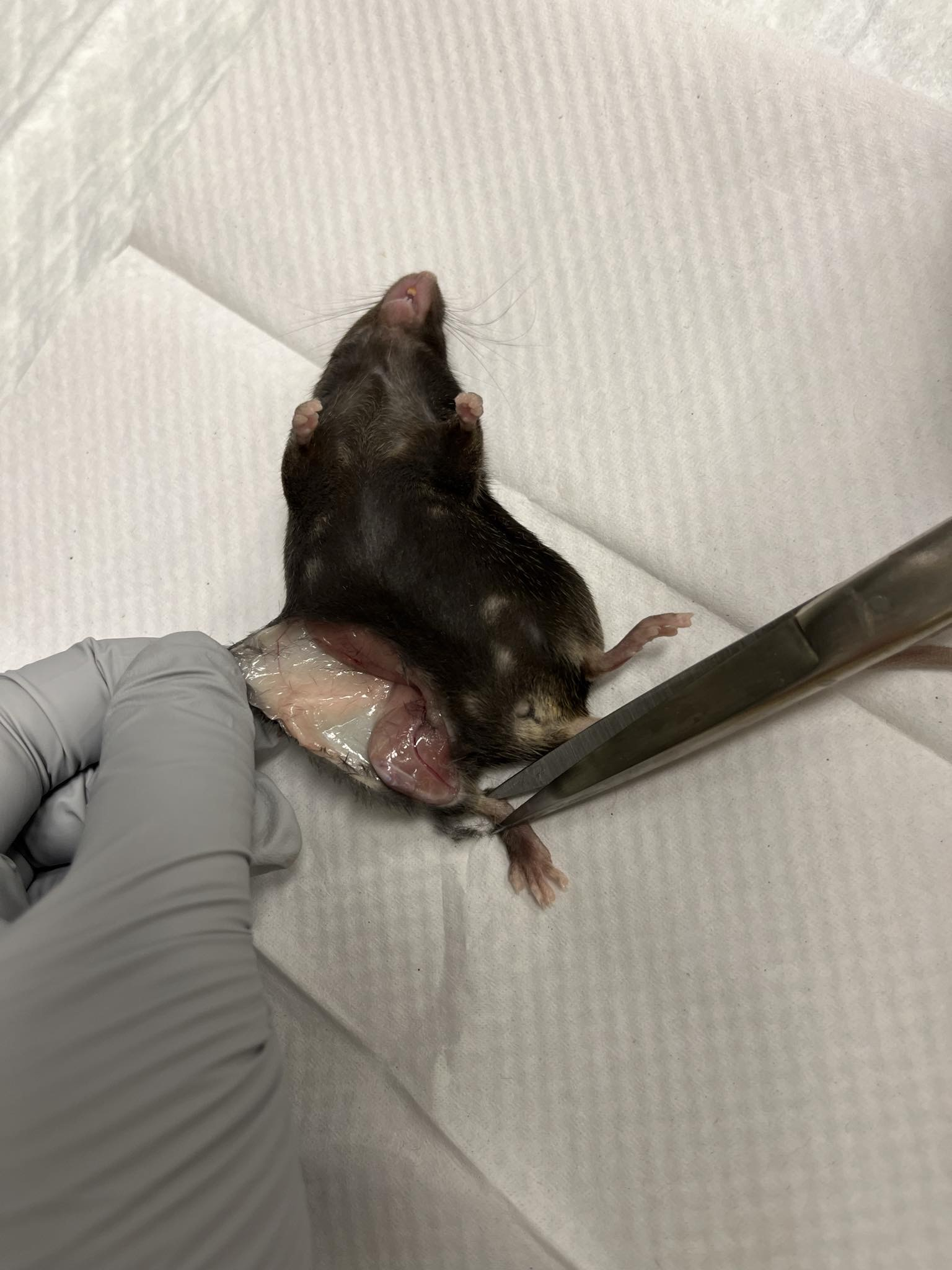

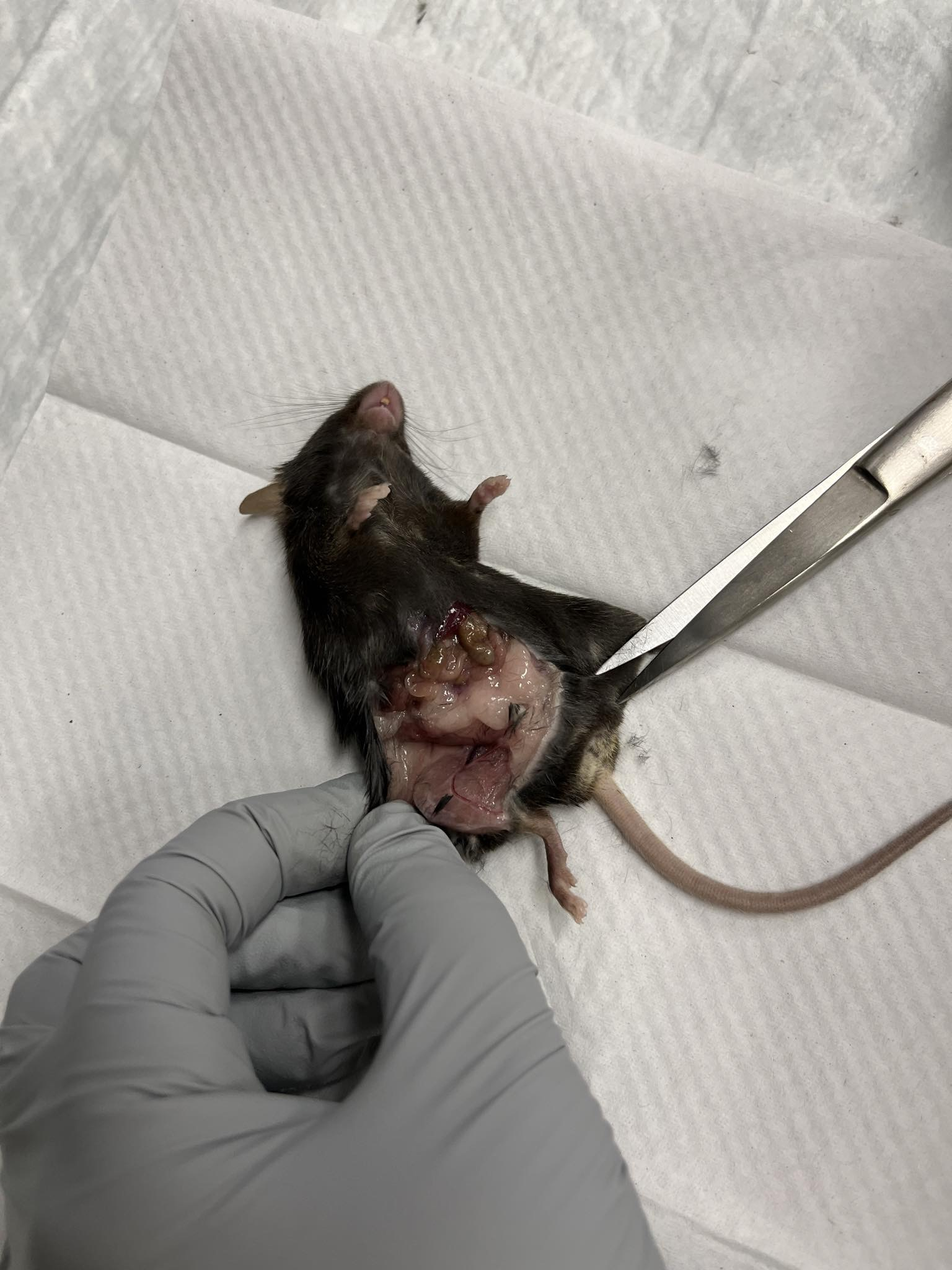


**Supplementary Figure 7: Subcutaneous fat accumulation in female mice.** During dissection, a fat accumulation was noticed in DYSF-R1925X all 3 female mice in their lower abdomen and upper legs. The 3 male mice did not present fat accumulation in this area. The mice were all aged between 21 and 24 weeks.
